# Improving GRN re‐construction by mining hidden regulatory signals

**DOI:** 10.1049/iet-syb.2017.0013

**Published:** 2017-12-01

**Authors:** Ming Shi, Weiming Shen, Yanwen Chong, Hong‐Qiang Wang

**Affiliations:** ^1^ State Key Laboratory for Information Engineering in Surveying, Mapping and Remote Sensing Wuhan University 129 Luoyu Road Wuhan 430079 People's Republic of China; ^2^ Machine Intelligence and Computational Biology Laboratory Institute of Intelligent Machines, Chinese Academy of Science PO Box 1130 Hefei 230031 People's Republic of China

**Keywords:** genetics, molecular biophysics, biology computing, singular value decomposition, sensitivity analysis, gene regulatory networks, gene expression, hidden regulatory signal mining, transcription factor, dictionary learning model k‐SVD, receiver operating characteristic curves

## Abstract

Inferring gene regulatory networks (GRNs) from gene expression data is an important but challenging issue in systems biology. Here, the authors propose a dictionary learning‐based approach that aims to infer GRNs by globally mining regulatory signals, known or latent. Gene expression is often regulated by various regulatory factors, some of which are observed and some of which are latent. The authors assume that all regulators are unknown for a target gene and the expression of the target gene can be mapped into a regulatory space spanned by all the regulators. Specifically, the authors modify the dictionary learning model, *k* ‐SVD, according to the sparse property of GRNs for mining the regulatory signals. The recovered regulatory signals are then used as a pool of regulatory factors to calculate a confidence score for a given transcription factor regulating a target gene. The capability of recovering hidden regulatory signals was verified on simulated data. Comparative experiments for GRN inference between the proposed algorithm (OURM) and some state‐of‐the‐art algorithms, e.g. GENIE3 and ARACNE, on real‐world data sets show the superior performance of OURM in inferring GRNs: higher area under the receiver operating characteristic curves and area under the precision–recall curves.

## 1 Introduction

The dynamic nature of gene regulatory networks (GRNs) plays critical roles in cellular activity and ultimately determines biological processes in a cell [1, 2]. A major issue in systems biology is to unveil the GRNs for comprehensively elucidating molecular mechanisms of cells and uncovering disease aetiology [3, 4, 5]. With the increasingly accumulated high throughput data, reverse‐engineering GRNs from transcriptomic data has become an important and cost‐effective approach for this issue [5]. Lots of efforts have been devoted to this issue from the community, e.g. the DREAM project (http://dreamchallenges.org/). However, untangling the comprehensive gene regulation networks (GRNs) is now still a challenging task due to the complexity of cellular regulatory system and limited knowledge about it in bioinformatics and computational biology [6, 7].

A number of computational approaches have been presented to reconstruct GRNs from transcriptomic data [8, 9, 10, 11, 12, 13, 14, 15, 16, 17]. In general, these GRN inference approaches fall into two categories: parameterised topology methods and unparameterised topology methods [16, 18, 19]. For the former, network topology is often parameterised using generative network models, such as Boolen networks [20], Gaussian graphical models (GGMs) [21], Bayesian networks [22], or Petri nets [23]. Earlier, Shmulevich *et al.* [24] introduced a probabilistic Boolean network model for GRN inference, which, as a probabilistic generalisation of standard Boolean networks, allows uncertainty in model selection and offers more flexibility in inferring GRNs compared with Boolean network models. Recently, Vasic *et al.* [25] extended the original probabilistic Boolean model for GRN inference by adding information theoretic rules. GGMs are another commonly used tool for GRN inference, especially for small‐ or median‐scale gene networks [26]. For example, Tian *et al.* [27] applied a graphical model to identify tissue‐specific gene regulations. While the GGMs‐based methods focus on inferring undirected biological networks, the Bayesian networks‐based methods are used to infer directed GRNs. Roy *et al.* [28] proposed to utilise the Bayesian networks to infer regulatory programmes for individual genes under a probabilistic constraint of module‐level organisation of regulatory networks. Siahpirani and Roy [29] recently further improved this Bayesian network‐based method by adding prior information of sequence‐specific motifs. However, most of the parameterised methods face two challenging problems when it comes to data sets with a large number of genes: (i) they can be very time‐consuming due to huge amounts of parameters being trained; (ii) a larger number of observations than usually available are required to reliably estimate network parameters [30]. Many efforts have been made to deal with these problems. For instance, Schafer *et al.* proposed to estimate the partial correlation matrix with the Moore–Penrose pseudoinverse of the sample correlation matrix, which is always irreversible due to limited number of samples available [31]. Wang *et al.* [32] proposed to utilise a regression approach for asymptotically efficient estimation of each entry of the precision matrix of the graphical model under a sparseness condition relative to the sample size. Considering that GRNs are scale‐free network and that hub genes play an essential role in gene regulation, Yu *et al.* [17] developed an improved GGM for GRN inference by incorporating prior information about hub genes.

The unparameterised topology methods mainly rely on measuring pair‐wise dependencies between genes based on linear or non‐linear correlation models. The reconstructed networks are also known as gene association networks [33, 34]. The Pearson correlation coefficient (PCC) is a commonly used rule for measuring the linear correlation between a pair of genes [35, 36, 37]. However, PCC have limited power in detecting complicated dependency patterns between genes [38]. Information theory‐based rules, e.g. mutual information and its various variants, have been extensively used to model the non‐linear dependencies between genes [39]. One drawback of standard mutual information rule per se cannot distinguish direct regulations from indirect regulations [12]. Conditional mutual information (CMI), as an extension of mutual information rule [40, 41], has been applied to distinguish direct regulations from indirect one [42]. However, CMI is often too stringent to objectively estimate regulatory relationships [38, 43, 44]. To alleviate the false‐negative problem caused by CMI, Margolin *et al*. [42] proposed to filter out the indirect regulations with the data procession inequality (DPI), while Meyer *et al*. [45] proposed to filter out indirect regulations with the maximum relevance/minimum redundancy feature selection. Recently, Zhang *et al.* [12] proposed a new statistic, namely conditional mutual inclusive information (CMII), which interrogates association between a pair of genes based on the Kullback–Leibler divergence by comparing the actual gene expression probability density and the postulated one when the possible link between the two genes is removed. However, in CMII, the imposed unimodal Gaussian distribution causes bias in GRN inference. Liu *et al.* [46] introduced the redundancy control strategy of information theory and combined with clustering technology for GRN inference. Running time is another crucial issue when applying information theory to data sets with a large number of genes, e.g. genome‐wide GRN reconstruction. Parallel computation frameworks have been introduced for improving computation efficiency of information theory‐based approaches [47, 48]. Liu *et al.* [49] proposed to simultaneously improve computation efficiency and eliminate the redundant regulations by incorporating CMI into local Bayesian networks. Glass *et al.* [50] proposed a message passing‐based framework for updating GRNs initialised by the Tanimoto similarity.

Another important line of unparameterised topology methods is to see pair‐wise dependence inference as a linear or non‐linear regression problem, namely, solving for the regulatory relationships by regressing the expression levels of target genes on known transcriptional factors (TF), despite the drawbacks of vulnerability to incomplete knowledge of the system and the statistical challenge of calibrating complex models to limited data. For time‐serious data, similar strategies, linear or non‐linear ODE models, were also used to elucidate gene regulatory interactions [51]. Many traditional regression algorithms have been applied [3]. Sparse linear regression, e.g. *l*_1_ or *l*_0_ regularised linear regression, has been recently used for selecting true regulators from a group of candidates as GRNs are a kind of sparse biological networks per se, i.e. a target gene is often directly regulated only by a small subset of regulators. For example, Geeven *et al.* [52] predicted TF–TF and TF–target gene interactions using LAsso models based on gene expression data in combination with DNA sequence data (GEMULA); Haury *et al.* [53] combined least angle regression with stability selection technology for improving the accuracy of inferred regulatory relationships. To address the non‐linear regulatory pattern problem, Huynh‐Thu *et al.* [54] proposed a tree‐based ensemble method (GENIE3) by wrapping linear regression model in a random forest framework, which relies on an interaction ranking rule, instead of regressed association values, and was the best performer in the DREAM5 network inference challenge.

However, the regression model‐based methods just ignore the influences from unknown or unobserved regulators. As we know, limited by human knowledge, there are a number of potential regulatory units still unknown, referred to as latent regulators (LRs) [3]. Ignoring these LRs necessarily degrades the performance of the regression models due to the incompletion of predictors. A natural way to approach the problem is to first discover as many LRs as possible from the expression matrix of genes. This is analogous to ‘signal restoration’ in the field of signal processing [55, 56, 57]. Dictionary learning is a new and promising approach for ‘signal restoration’, which essentially finds the subspace or dictionaries where the observed data (e.g. natural patches, audio segment, or even gene expression data) lies and determines the efficient representations of the data in the subspace. Generally speaking, sparsity constraints are keys to most of the algorithms of dictionary learning: they enforce the identification of the most important causes of the observed data and favour an accurate representation of the relevant information. Dictionary learning has been widely applied in signal processing areas for image denoising, audio processing, as well as classification, proving that it outperformed traditional wavelet analysis in discovering hidden signals [58, 59, 60, 61].

To our knowledge, using dictionary learning to reconstruct regulatory networks has not been explored. We here propose to improve GRN inference by utilising dictionary learning to interrogate hidden regulatory signals, including TFs, non‐coding RNA, and methylation, and remove their influences. An assumption is made that the expression profiles are shaped collectively by a handful of regulators, known or latent, in a weighted linear way and these regulators can be discovered by mapping gene expression data into a regulatory space spanned by the regulators. Finally, with the resulting regulators, a given TF can be predicted to be a real regulator for a target gene via a confidence score that measures the expression similarity between the TF and the resulting regulators. We applied the model to analyse synthetic as well as real gene expression data about two model organisms (*Escherichia coli* and *Saccharomyces cerevisiae*) and demonstrated the superior performance of the proposed model for inferring GRNs.

## 2 Methods

### 2.1 A global model for gene regulatory system

**Fig. 1:**
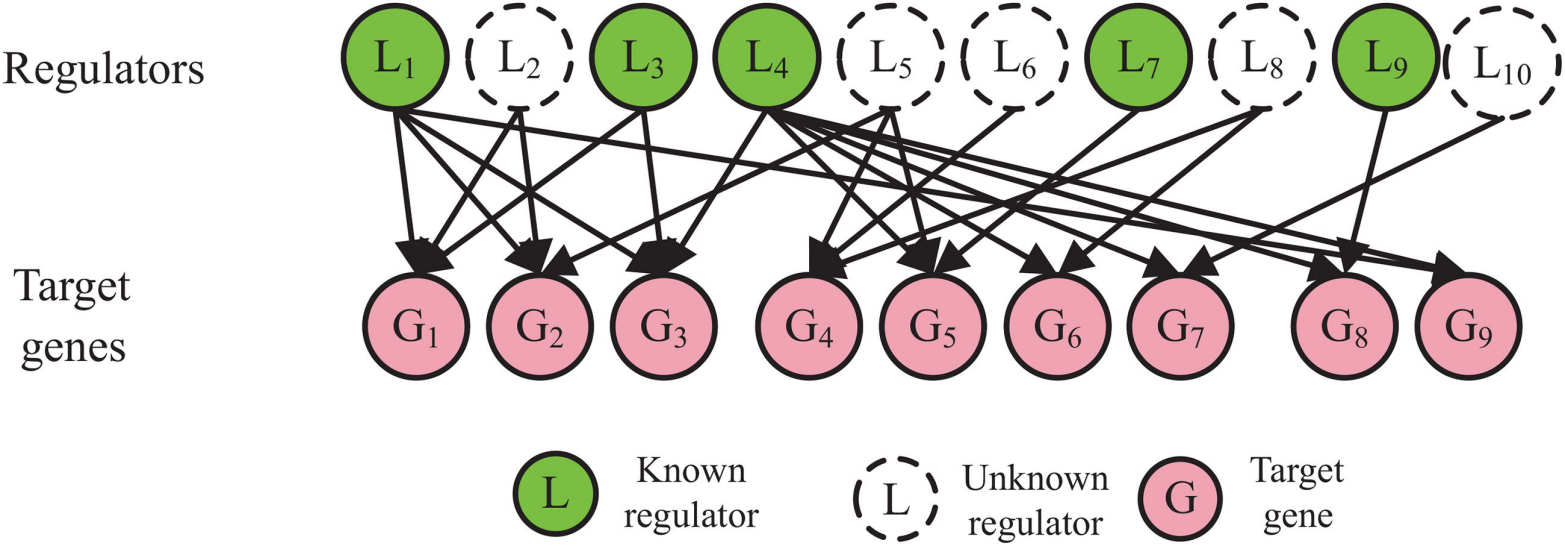
Global gene regulatory model for target genes with known and unknown regulators

In biological organisms, gene expression can be controlled and regulated by various factors, such as transcription factors, microRNAs, or even their own methylation status. Restricted by technology, not all of them are known or detectable in a biological experiment, and only few thousands of TFs and microRNAs are known by now. Keeping this in mind, we assume that there are many unknown regulators that involve in shaping the expression level of target genes but that are not detected, as depicted in Fig. 1. These regulators can be comprehensively discovered using dictionary learning, a data mining model widely applied in pattern recognition and signal processing fields. Mathematically, we represent a gene expression matrix ***Y*** in the following formula

(1) Y=DX+ε

where ***D*** represents the expression matrix of the regulators, ***X*** represents a sparse coefficient matrix of the regulators on target genes, and ε represents random noise subjecting to an i.i.d*.* Gaussian distribution with the mean of zero. The regulators could be known or latent. To mine the regulators for a given ***Y***, we need to find both proper ***D*** and ***X***, i.e. to solve (1) means to simultaneously optimise ***D*** and ***X*** given ***Y***.

### 2.2 Simultaneously learning known and hidden regulator signals

We approximate the solutions of (1) using the following optimisation problem:

(2) $$(\tilde{D},\tilde{X})=\arg\min_{D,X}{∥Y-DX∥}_{2}^{2}\;s.t.\;{∥x_{i}∥}_{0}\leq t_{i},\;i=1,\ldots ,p$$

where ***x*** *_i_* is the *i* th column of ***X***, *p* the number of the target genes, and $t_{i}$ a small positive constant referred to as sparsity parameters. Suppose *n* samples, we have $D\in R^{n\times l}$ and $X\in R^{l\times p}$ where *l* is the number of LRs. The minimisation problem (2) is not convex on both ***D*** and ***X*** together and solving this problem is NP‐hard because of the *l*_0_ ‐norm constraint. Currently, there are no immediate solutions available for the optimisation problem. Practically, it is desirable to find local minima for such optimisation problems [62]. We developed a modified *k* ‐SVD algorithm (Supplementary material) to approximate the global optimum solution. The algorithm iterates between the sparse approximation and the dictionary learning steps until convergence. A proof for the convergence of the optimisation algorithm is given in Supplementary material.

### 2.3 Inferring regulatory relationships from signal dictionary

**Fig. 2:**
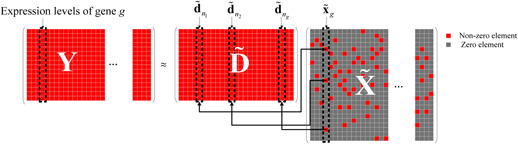
Illustration of regulatory relationships inference

As described above, the *g* th column of $\tilde{X}$, ${\tilde{x}}_{g}$, specifies the regulatory coefficients of all regulators on the target gene *g*, whose non‐zero elements represent a regulatory relationship. Suppose that the vector ${\tilde{x}}_{g}$ has *n_g_* non‐zero elements whose subscripts are $f(1),f(2),\ldots ,f(n_{g})$, respectively, it can be inferred out that for gene *g*, there could have *n_g_* regulators whose expression levels are the *n_g_* columns of $\tilde{D}$, $\text{Rd}=\left\{{\tilde{d}}_{f(1)},{\tilde{d}}_{f(2)},\ldots ,{\tilde{d}}_{f(n_{g})}\right\}$, as illustrated in Fig. 2.

Considering all possible regulators, known or unknown, detectable or undetectable, are encapsulated in Rd for target gene *g*, one can intuitively determine if a known regulator specifically regulate and mediate the expression of gene *g* by matching with Rd. Suppose a known transcription factor tf whose expression levels are ***d***_tf_ and Γ represents the distribution of the correlation coefficients of tf with the resulted regulators, e.g. linear Pearson correlation or non‐linear Spearman correlation. We estimate the regulation confidence of tf on target gene *g* as follows:

(3) $$CS_{\text{tf}\to g}=C_{\Gamma }^{-1}\left(\alpha \right)$$

where *C* ^−1^ represents the inverse (i.e*.* quantile) function of Γ, and 0≤α≤1 represents a given quantile point. One can vary *α* for a proper estimation of regulation confidence, of which larger values lead to more sensitive results. We take *α*  = 1 to calculate the confidence score for a high recognition sensitivity. Finally, we call the transcription factor tf to be a regulator of the target gene *g*, if the following equation holds

(4) $$CS_{\text{tf}\to g}\geq T$$

where 0 < *T* *≤*  1 is a confidence cut‐off value and was set to be 0.8 for a high confidence of regulatory relationship.

### 2.4 Measures for evaluation

To evaluate the power of mining hidden regulatory signals, two measures, i.e. recovery rate and precision, were used. Suppose *k* true regulators in background networks, $d_{1},d_{2},\ldots ,d_{k}$ and *l* recovered regulators by OURM, ${\tilde{d}}_{1},{\tilde{d}}_{2},\ldots ,{\tilde{d}}_{l}$, we define the recovery rate and precision as follows:

(5) $$\begin{array}{rl} \text{Recovery}\;\;\text{rate} & =\frac{1}{k}\sum_{i=1}^{k}I\left(\sum_{j=1}^{l}I\left(\left|\rho \left(d_{i},{\tilde{d}}_{j}\right)\right|\geq 0.99\right)>0\right)\times 100\%\\ \text{Precision} & =\frac{1}{l}\sum_{j=1}^{l}I\left(\sum_{i=1}^{k}I\left(\left|\rho \left({\tilde{d}}_{j},d_{i}\right)\right|\geq 0.99\right)>0\right)\times 100\% \end{array}$$

where I(⋅) is an indicator function valued 1 if the statement is true and 0 otherwise.

Additionally, two measures, i.e. area under the receiver operating characteristic (AUROC) curve and area under the precision–recall (AUPR) curve, were adopted to assess the performance of different algorithms in recognising regulatory relationships. Receiver operating characteristic (ROC) curves plot false‐positive rates against true‐positive rates, while precision–recall (PR) curves plot precisions against recalls.

### 2.5 Data sets

We evaluated the proposed method on both simulation data and real gene expression data. The simulation data were generated as follows: assume total *k*  = 50 regulators, *p* *=*  1500 target genes, and *n* samples. First, we simulated the expression profiles of the *k* regulators in the *n* samples $D\in R^{n\times k}$ by randomly sampling from a standard normal distribution. Second, we created background networks with a connection matrix $X\in R^{k\times p}$, where non‐zero elements represent a regulatory relationship between the corresponding regulator and target gene and were sampled from a standard normal distribution. Considering the scale‐free property of natural GRNs, we let 50, 25, 15, and 10% of target genes be regulated by 2, 3, 4, or 5 regulators, respectively. Third, we synthesised the expression profiles of target genes Y by ***Y*  = *DX*** plus Gaussian noise. To mimic different levels of noise, the signal‐to‐noise ratio (SNR) was varied among SNR = 10, 15, 20, 25, 30, or 40. Meanwhile, to mimic the influence of sample size, the number of samples was varied among *n*  = 20, 30, 50, 100, or 200. So, there are totally 30 (=6 × 5) scenarios containing 6 different intensities of noise and 5 different sample amounts. To avoid randomness, in each setting of (*n*, *k*, SNR), we randomly generated 20 data sets and evaluated algorithms using average results.

We also downloaded a synthesised non‐linear gene expression data set as well as two real gene expression data sets from DREAM5 (http://www.the‐dream‐project.org/). The synthesised data contains the synthesised expression profiles of 1643 genes, of which 195 are TFs, in 805 chips, and was produced from a real regulatory network of *E. coli* consisting of 4012 experimentally verified TF–TG gene interactions. For the detail of the generation procedure for the synthesised data set, refer to the literature [6, 63]. The two real data sets are about two model organisms *E. coli* and *S. cerevisiae*, respectively: one consists of the expression profiles of 4511 target genes and 334 TFs in 805 samples and the other the expression profiles of 5950 genes, of which 333 are TFs across 536 chips. For the two real data sets, 2066 and 3940 experimentally verified TF–TG gene interactions were obtained from RegulonDB database [64] and Zhu *et al.* [65], for algorithm evaluation in this study, respectively.

## 3 Results

### 3.1 Evaluation on simulation data

We first evaluated the performance of GRN reconstruction on the simulated data. For comparison, we also applied four previous methods, GENIE3 [54], CLR [14], ARACNe‐AP [15], and ARACNE [42, 66], to analyse the simulation data. The DPI parameter in ARACNE, which marked as ‘eps’, was varied among 0, 0.05, 0.15, and 0.2 for fully comparison. Table 1 lists the results of OURM and the four previous methods on the simulated data sets of *n*  = 20. From Table 1, the following conclusions can be drawn: first, generally speaking, the proposed algorithm achieves the highest average AUROCs and AUPRs across almost all data sets (shown in bold) with different settings of *l*. Second, large *l* s, e.g. not less than the number of regulators in the network (50), led to high average AUROC and average AUPR. These results suggest that a large value of *l* (e.g. larger than the number of regulators) can be recommended for accurate GRNs reconstruction in practice.

**Table 1:** Performances of different GRN inference methods on the simulated data of *n*  = 20

Data set	Method	AUROC		AUPR	
(*n*, SNR)		Mean	Std	Mean	Std
(20, 10)	GENIE3	0.811	0.0049	0.538	0.0077
	CLR	0.810	0.0029	**0.577**	0.0042
	ARACNe‐AP	0.626	0.0145	0.137	0.0537
	ARACNE (eps = 0)	0.780	0.0024	0.574	0.0048
	ARACNE (eps = 0.05)	0.804	0.0045	0.554	0.0046
	ARACNE (eps = 0.1)	0.814	0.0050	0.553	0.0052
	ARACNE (eps = 0.2)	0.818	0.0043	0.552	0.0053
	OURM (*l* = 25)	0.749	0.0073	0.186	0.0112
	OURM (*l* = 50)	**0.857**	0.0049	0.430	0.0101
	OURM (*l* = 100)	0.855	0.0041	0.478	0.0089
	OURM (*l* = 150)	0.849	0.0042	0.494	0.0099
(20, 20)	GENIE	0.820	0.0051	0.561	0.0061
	CLR	0.819	0.0036	0.598	0.0053
	ARACNe‐AP	0.642	0.0061	0.186	0.0377
	ARACNE (eps = 0)	0.791	0.0019	0.610	0.0046
	ARACNE (eps = 0.05)	0.810	0.0035	0.574	0.0060
	ARACNE (eps = 0.1)	0.820	0.0047	0.572	0.0068
	ARACNE (eps = 0.2)	0.826	0.0041	0.572	0.0073
	OURM (*l* = 25)	0.733	0.0092	0.168	0.0129
	OURM (*l* = 50)	0.904	0.0049	0.540	0.0121
	OURM (*l* = 100)	**0.908**	0.0051	0.598	0.0101
	OURM (*l* = 150)	0.906	0.0039	**0.627**	0.0117
(20, 30)	GENIE	0.819	0.0051	0.560	0.0078
	CLR	0.817	0.0044	0.595	0.0069
	ARACNe‐AP	0.641	0.0132	0.192	0.0703
	ARACNE (eps = 0)	0.789	0.0055	0.605	0.0075
	ARACNE (eps = 0.05)	0.809	0.0061	0.567	0.0111
	ARACNE (eps = 0.1)	0.819	0.0046	0.565	0.0106
	ARACNE (eps = 0.2)	0.825	0.0062	0.564	0.0110
	OURM (*l* = 25)	0.734	0.0078	0.168	0.0112
	OURM (*l* = 50)	0.915	0.0059	0.569	0.0150
	OURM (*l* = 100)	**0.922**	0.0052	0.629	0.0132
	OURM (*l* = 150)	0.920	0.0041	**0.658**	0.0099

**Fig. 3:**
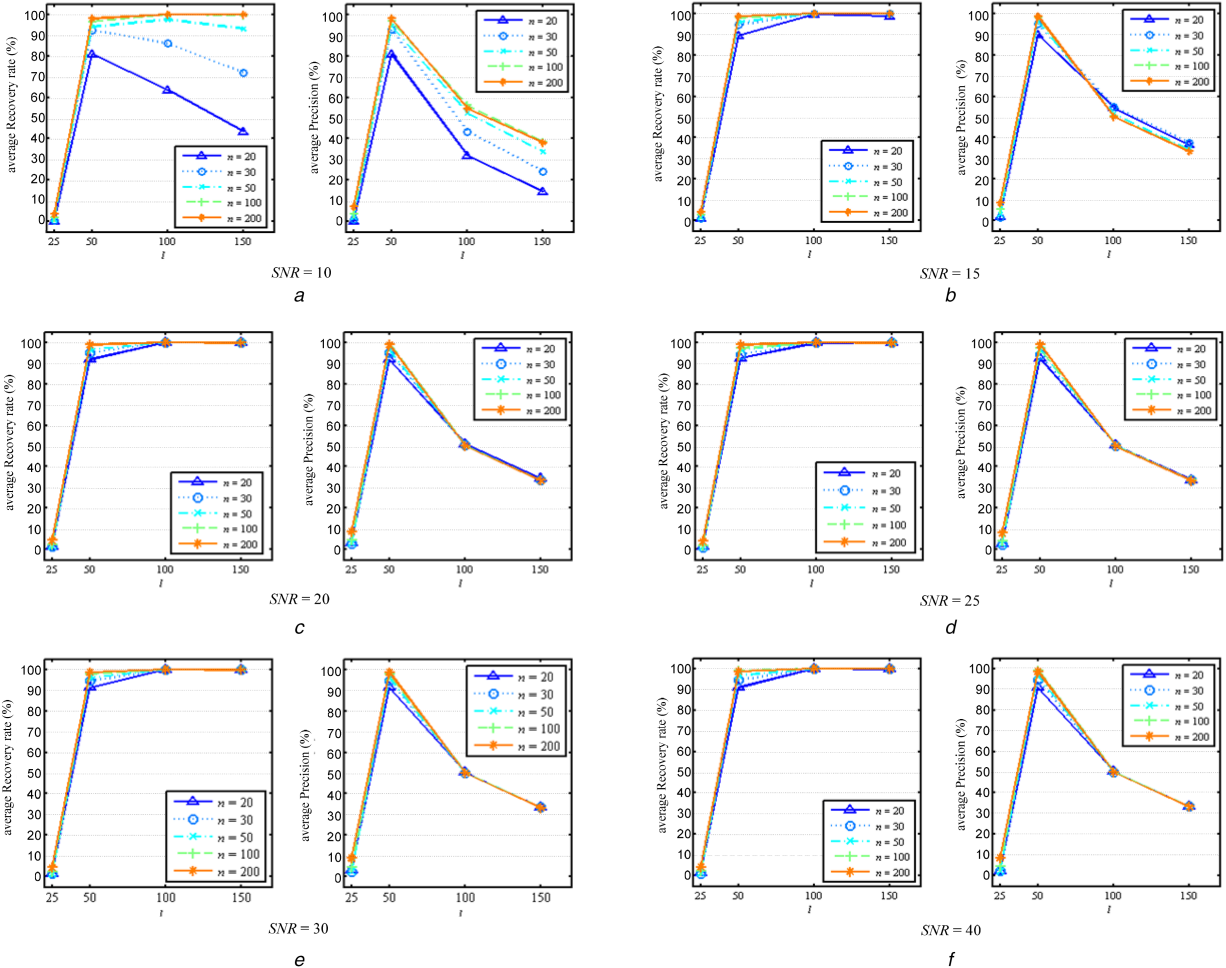
Average recovery rates and precisions of the proposed approach on simulated data *(a)* SNR = 10, *(b)* SNR = 15, *(c)* SNR = 20, *(d)* SNR = 25, *(e)* SNR = 30, *(f)* SNR = 40

The power of the proposed method in recovering hidden regulatory signals on the simulation data was subsequently calculated. Considering the parameter *l* is an important parameter which specifies the number of hidden signals to be mined, we varied the parameter *l*  = 25, 50, 100, 150 to learn hidden regulatory signals. Fig. 3 shows the changes of the average recovery rates as well as precisions with *l* on all the simulation data scenarios.

From Figs. 3 *a*–*f*, it can be seen that when the value of parameter *l* is set to be 50, i.e. the number of regulators in the networks, both average recovery rates and average precisions are larger than 80%, irrespective of sample size and noise level. When *l* is larger than the number of regulators, e.g. *l*  = 100 and 150, the average recovery rates continued to increase up to 100%, but the average precisions began to drop: the average recovery rates approximate 100%, indicating that almost all of the 50 regulators are successfully recovered in the *l* mined signals, while the average precisions approximated to 50/*l*, e.g. 50% with *l*  = 100 and 30% with *l*  = 150, suggesting a decrease in average precisions with increasing *l*. In contrast, when *l* is less than the number of regulators, e.g. *l*  = 25, both average recovery rates and average precisions are always in a lower level, e.g. lower than 10% (Figs. 3 *a*–*f*). These results indicate that the value of *l* around the number of regulators tends to a best performance of recovering hidden regulatory signals and larger values will be preferred to a smaller value in practice.

Fig. 3 also reveals that when the data is highly noisy, the number of samples (*n*) also took substantial impact on the regulatory signal recovery ability of OURM as expected (Fig. 3 *a*). From Fig. 3 *a*, it can be clearly seen that both average recovery rates and precisions varied largely from *n*  = 20 to 200, and the change amplitude enlarged as *l* increases. However, high SNRs dwarf the improvements of average recovery rates and average precisions by increased numbers of samples, as shown in Figs. 3 *b*–*f*. This implies that an increased number of samples is preferred for overcoming noise contamination of data.

**Fig. 4:**
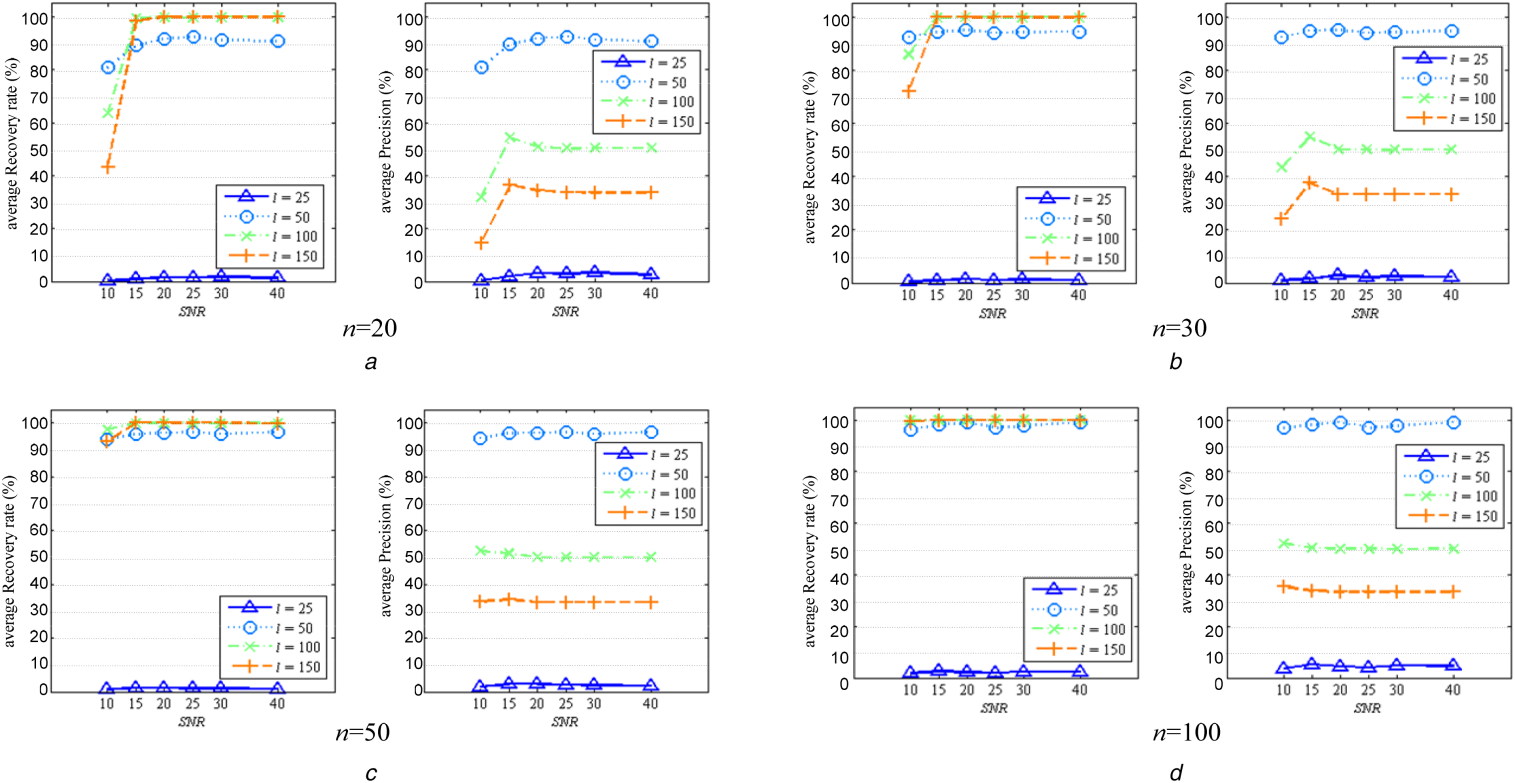
Influence of SNR on average recovery rates and average precisions on simulation data *(a) n*  = 20, *(b) n*  = 30, *(c) n*  = 50, *(d) n*  = 100

For clearly observing the influence of noise, we further examined the changes of the average recovery rates and precisions with the level of SNR in each data scenario of *l* and *n*, as shown in Fig. 4. From Fig. 4, we can clearly see that average recovery rates and precisions generally increase as SNR increases in all data scenarios as expected, irrespective of *l* and *n*. The tendency is more obvious in the cases of smaller sample sizes, e.g. *n*  = 20, 30, and larger values of *l*, e.g. *l*  = 50, 100, 150, as shown in Figs. 4 *a* and *b*.

### 3.2 Application to synthesised non‐linear gene expression data

We next evaluated the performance of the proposed method in GRN reconstruction on the synthesised non‐linear gene expression data. Results are shown in Table 2. Considering the network size in this scenario, i.e. total 1643 genes, *l* was set to be 150, 200, 300, 400, or 500. Generally speaking, as can be seen in the table, AUROC and AUPR scores first increase with *l* changing from 150 to 200 and then both rapidly go down with *l* becoming larger. More interestingly, the optimal value of *l* seems to be around 200, which approximates the number of transcription factors of the background network, i.e. 195, confirming the guidance of choosing *l* above. For comparison, we also applied four previous methods, GENIE3 [54], CLR [14], ARACNe‐AP [15], and ARACNE [42, 66], to analyse the simulation data and the previous methods. The DPI parameter in ARACNE, i.e. ‘eps’, was varied among 0, 0.05, 0.1, and 0.2 for fully comparison with the proposed method. From this table, we can clearly see that our method with *l*  = 200 achieves the highest AUROC scores (0.826) among these methods.

**Table 2:** Comparison results of different methods on synthesised non‐linear and real‐world data sets

Method	Synthesised non‐linear data		*E. coli*		*S. cerevisiae*	
	AUROC	AUPR	AUROC	AUPR	AUROC	AUPR
GENIE3	0.815	**0.284**	**0.717**	**0.0211**	0.529	0.0031
CLR	0.743	0.226	0.587	0.0112	0.524	0.0022
ARACNe‐AP	0.682	0.156	0.566	0.0061	0.516	0.0002
ARACNE (eps = 0)	0.642	0.243	0.539	0.0102	0.505	0.0029
ARACNE (eps = 0.05)	0.739	0.199	0.515	0.0074	0.488	0.0019
ARACNE (eps = 0.1)	0.755	0.192	0.597	0.0078	0.505	0.0021
ARACNE (eps = 0.2)	0.757	0.191	0.617	0.0080	0.530	0.0022
OURM (*l* = 150)	0.815	0.227	0.679	0.0042	0.530	0.0021
OURM (*l* = 200)	**0.826**	0.232	0.682	0.0043	0.535	0.0022
OURM (*l* = 300)	0.715	0.117	0.627	0.0193	0.530	0.0031
OURM (*l* = 400)	0.722	0.132	0.653	0.0124	0.520	0.0030
OURM (*l* = 500)	0.709	0.129	0.652	0.0111	**0.537**	**0.0033**

### 3.3 Applications to two real gene expression data

We also evaluated our approach based on two real gene expression data sets, *E. coli* and *S. cerevisiae*. Considering the network sizes of the two model organisms and the complexity of gene regulation in real world, *l* was varied among 150, 200, 300, 400, and 500 in the experiments. Experimental results of OURM and the four previous methods on these two data sets are listed in Table 2, showing that OURM achieved comparative performance with the three previous methods on both of the data sets and specially, on the *S. cerevisiae* data, OURM achieves the highest AUROC and AUPR among the four algorithms. For the *E. coli* data, our method achieved its highest AUROC (0.682) and AUPR (0.0193) when *l*  = 200 and 300, respectively, while for the *S. cerevisiae* data, our method achieved its highest AUROC (0.537) and AUPR (0.0033) when *l*  = 500. This could imply that more LRs are involved in the *S. cerevisiae* data than in the *E. coli* data, suggesting a more complex molecular system in eukaryotic organisms than in prokaryotic organisms.

**Fig. 5:**
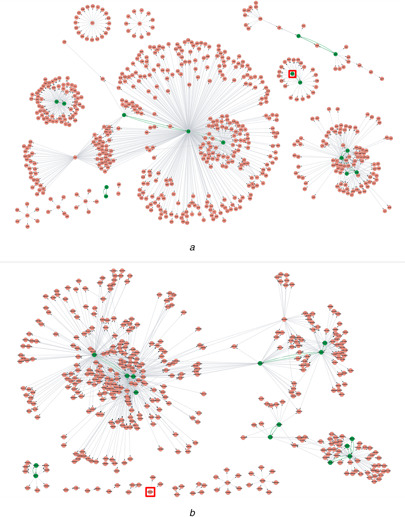
Inferred networks of OURM on real gene expression data collected from E. coli and S. cerevisiae. The transcription factor ‘gadX’ and ‘YBR240C’ are each surrounded by a square. The location of the genes involved in feedback loops are marked in green ***(a)*** Inferred network for *E. coli*, **(*b)*** Inferred network for *S. cerevisiae*

**Fig. 6:**
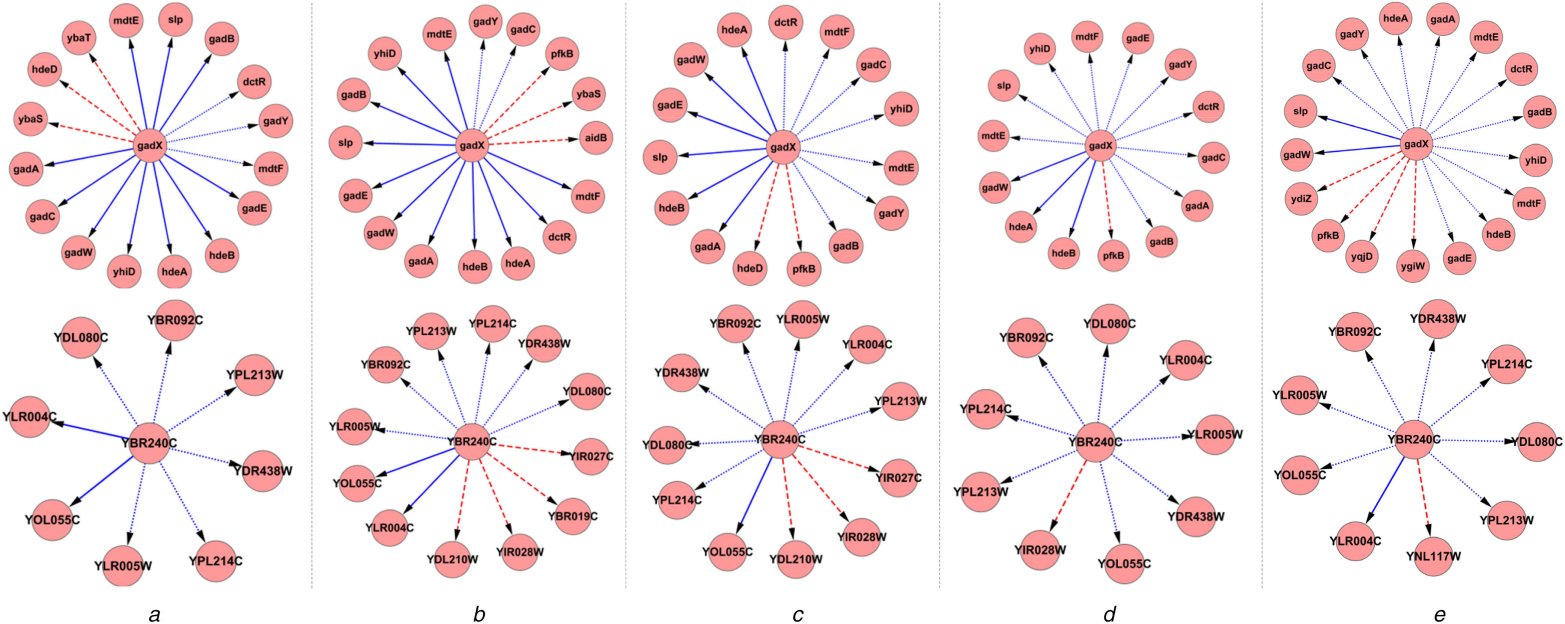
Inferred target genes of gadX and Y240C by OURM, GENIE3, CLR, ARACNe‐AP, and ARACNE (eps = 0). Solid arrows represent true regulatory interactions correctly recognised, dotted arrows represent the missing regulatory interactions, and dashed arrows represent the wrongly inferred regulations ***(a)*** Inferred target genes of gadX and Y240C by OURM, ***(b)*** Inferred target genes of gadX and Y240C by GENIE3, ***(c)*** Inferred target genes of gadX and Y240C by CLR, ***(d)*** Inferred target genes of gadX and Y240C by ARACNe‐AP, ***(e)*** Inferred target genes of gadX and Y240C by ARACNE

Fig. 5 shows the structures of the GRNs inferred by OURM on the two real gene expression data sets. The network (Fig. 5 *a*) of *E. coli* consists of 633 genes (including 27 TFs) and 957 regulatory links. The network (Fig. 5 *b*) of *S. cerevisiae* consists of 818 regulations among 436 genes, 27 of which are TFs. Besides, we found that there are totally nine feedback loops in the inferred *E. coli* network (Fig. 5 *a*) and ten in the inferred *S. cerevisiae* network (Fig. 5 *B*). These inferred feedback loops are marked in green in the subfigures. For fair comparison, we trimmed the GRNs inferred by the other three previous methods to have the same number of edges, i.e. 957 for *E. coli* and 818 for *S. cerevisiae*, by taking proper thresholds of regulation coefficients. To exemplifying the differences in GRN inference abilities among the five algorithms OURM, GENIE3, CLR, ARACNe‐AP, and ARACNE (eps = 0), we illustrate the inferred target genes of two transcription factors, i.e. gadX from *E. coli* and YBR240C from *S. cerevisiae*, in Figs. 6 *a*–*e*. The former has been biologically recognised as an acid resistance regulon transcriptional activator [67], while the latter is involved in transcriptional regulation of thiamine biosynthesis [68]. From this figure, we can clearly see that OURM successfully recognised 10 of all the 13 experimentally verified targets of gadX, which are more than those by CLR (6), ARACNe‐AP (3), and ARACNE (2) though slightly <11 (GENIE3). For the transcription factor YBR240C, OURM successfully recovered most known target genes (2/8) and wrongly identified no target genes compared with the four previous methods. These results demonstrated the superior performance of OURM in inferring gene regulations on real gene expression data sets.

## 4 Discussions and conclusions

We have proposed a new computational method, OURM, for reverse engineering GRNs based on dictionary learning. The method factorised a transcriptomics data matrix of targets into a regulatory space. A modified version of *k* ‐SVD was developed for mining hidden regulatory structures between regulators and target genes, and a new statistic CS was then formulated for measuring regulatory relationships. Experimental results on simulation data and real‐world data sets demonstrated the effectiveness and efficiency of OURM for GRN inference.

Most existing models do not consider LRs often resulting in a degraded performance of GRNs inference. In the proposed method, we discovered the hidden regulator signals using dictionary learning followed by quantifying regulatory relationships. The recovery power was verified on the simulation data. On the other hand, we also noticed that the power can be influenced by the parameter *l*. Future works will be focused on the guidance of choosing the parameter in practice and evaluations on more real‐world data sets.

## Supporting information

Supplementary Data
