# Supplementary material for: Improving GRN re‐construction by mining hidden regulatory signals
Source: IET Syst Biol. 2017 Dec 1;11(6):174–81. doi: 10.1049/iet-syb.2017.0013 (PMC8687237; doi:10.1049/iet-syb.2017.0013)
Supplement: Supplementary file 1 — Supplementary Data [file SYB2-11-174-s001.docx]

1. *The flow chart of dictionary learning algorithm in OURM.*

| **Algorithm( Dictionary Learning Algorithm)** |
| --- |
| **Input** |
| Gene expression matrix , where *n* is the number of samples and *p* is the number of genes  Expression level of a transcription factor *tf*: **d***_tf_*  The number of regulators that regulate the expression of gene *i*: *t_i_*(*i*=1,2,…,*p*)  The number of signals to be mined: *l* |
| **Output** |
| Learned dictionary with each column corresponding to a mined signal or latent regulator (LR).  Coefficient matrix  for sparse representation of **Y** using$\tilde{\mathbf{D}}$. |
| **Initialization**  Set , *i.e.* the first *l* columns of gene expression matrix **Y**, $\mathbf{X}^{(0)}=\mathbf{0}\in R^{l\times p}$, *S*=1, where **Y**(1:*l*) represents the 1st to *l-th* column of matrix **Y**. |
| **Step-1.** (Sparse coding) Use any pursuit algorithm, *e.g.* Orthogonal Matching Pursuit (OMP), to compute the representations vectors **x***_i_* for the expression vector of gene *i*, *i.e.*$y_{i}$.  *i*=1,2,…,*p*, $\min_{\mathbf{x}_{i}} \left\{\left\Vert y_{i}-\mathbf{D}^{(S-1)}\mathbf{x}_{i}\right\Vert_{2}^{2}\right\} \text{subject} \text{to} \left\Vert\mathbf{x}_{i}\right\Vert_{0}\leq t_{i}$ Then replace the *i*-th column of $\mathbf{X}^{(S-1)}$ with **x***_i_*with *i*=1, 2,…,*p*.  **Step-2.** (Codebook Update Stage) For each column *j*=1,2,…,*l* in $\mathbf{D}^{(S-1)}$, update it by   - Define the group of examples that use this atom $\omega_{j}$=$\left\{\gamma\vert1\leq\gamma\leq p,\mathbf{X}^{(S-1)}(j,\gamma)\neq0\right\}$ with $\mathbf{X}^{(S-1)}(j,\gamma)$ represents the (*j*, $\gamma$)-th entity of matrix $\mathbf{X}^{(S-1)}$ - Compute the overall representation error matrix without using this atom, $\mathbf{E}_{j}=\mathbf{Y}-\sum_{m\neq j} \mathbf{D}^{(S-1)}(:,m)\cdot\mathbf{X}^{(S-1)}(m,:)$ - Restrict $\mathbf{E}_{j}$by choosing only the columns corresponding to $\omega_{j}$, and obtain $\mathbf{E}_{j}^{R}=\mathbf{E}_{j}(:,\omega_{j})$ that implies a selection of error columns corresponds to genes that use the atom $\mathbf{D}^{(S-1)}(:,j)$ - Apply SVD $\mathbf{E}_{j}^{R}=\mathbf{U\Delta V}^{T}$. Replace the *j-th* column in $\mathbf{D}^{(J-1)}$ with the first column of **U**. Replace the coefficient vector $\mathbf{X}^{(S-1)}(:,j)$ to be the first column of **V** multiplied by $\mathbf{\Delta}(1,1)$ - Set$\mathbf{D}^{(S)}=\mathbf{D}^{(S-1)},\mathbf{X}^{(S)}=\mathbf{X}^{(S-1)}$, as well as *S*=*S*+1   **Step-3.** If *S*>200 or $\left\Vert\mathbf{Y}-\mathbf{D}^{(S)}\mathbf{X}^{(S)}\right\Vert_{2}^{2}\leq1e^{-6}$, return to step-1  **Step-4.** Set $\tilde{\mathbf{D}}=\mathbf{D}^{(S)},\tilde{\mathbf{X}}=\mathbf{X}^{(S)}$ |

1. *The proof of convergence of representation errors.*

**Theorem** In the dictionary learning algorithm, the sequence $\left\{\left\|\mathbf{Y}-\mathbf{D}^{(S)}\mathbf{X}^{(S)}\right\|_{2}^{2}\right\}$ with *S*=1,2,…is convergent.

**Proof**: In Step-1 of the *flow chart*, the representation error, *i.e.* the Frobenius norm of$\mathbf{Y}-\mathbf{D}^{(S-1)}\mathbf{X}^{(S-1)}$, is lowered by the update of column vectors in $\mathbf{X}^{(S-1)}$ step by step. In Step-2, the representation error is further lowered in SVD step. Thus, we get the conclusion that the number sequence $\left\{\left\|\mathbf{Y}-\mathbf{D}^{(S)}\mathbf{X}^{(S)}\right\|_{2}^{2}\right\}$ is monotonically decreasing.

In consideration that $\left\|\mathbf{Y}-\mathbf{D}^{(S)}\mathbf{X}^{(S)}\right\|_{2}^{2}$ for all S>0, so the sequence is bounded.

With the analysis above, the sequence $\left\{\left\|\mathbf{Y}-\mathbf{D}^{(S)}\mathbf{X}^{(S)}\right\|_{2}^{2}\right\}$is convergent.
